# Association between APOE ε4 Genotype and Memory Impairment in Elderly with Normal Global Cognitive Assessment

**DOI:** 10.3390/diagnostics5040615

**Published:** 2015-12-15

**Authors:** Yuda Turana, Yvonne Suzy Handajani, Nelly T Widjaja

**Affiliations:** 1 Department of Neurology, Faculty of Medicine, Atma Jaya Catholic University of Indonesia, Jl. Pluit Raya No. 2, North Jakarta, Jakarta 14440, Indonesia; 2 Center of Health Research, Atma Jaya Catholic University of Indonesia, Jl. Pluit Raya No. 2, North Jakarta, Jakarta 14440, Indonesia; E-Mails: ivonnes_su@yahoo.com (Y.S.H.); nt_widjaja@yahoo.com (N.T.W.)

**Keywords:** APOE, cognitive, early detection, elderly, memory

## Abstract

Aim: Early prediction using cognitive evaluation tools that are less influenced by education level is beneficial for dementia screening. This study investigated the relationship between Word List Memory Immediate Recall (WLM IR) and the Saving Score (SS) with having the APOE ε4 risk allele in the elderly with normal global cognitive assessment. Methods: A cross-sectional study on 105 subjects ≥60 years with normal MMSE scores who met inclusion criteria. Memory impairment (MI) if: WLM IR score on the third trial <8 or an SS score <80%. Results: The majority of the subjects were female (68.6%), 65 ± 7.1 years, had undertaken formal education for <6 years (56.2%), had MI (81%), and the APOE ε4 genotype was detected in 24.8% of subjects. There was a significant relationship between APOE ε4 and lower WLMIR (*p* = 0.02, OR 7.92, CI 95% (1.00–62.38)). Conclusions: WLM IR score is lower in elderly people with the APOE ε4 despite their normal global cognitive assessment results, and these scores were not influenced by education level. Further research needs to confirm that the WLM IR can be used to screen for early dementia.

## 1. Introduction

Early detection of Alzheimer Dementia (AD) is very substantial for management’s efficacy [1]. The main focus in uncovering degenerative process in the brain has been which reliable instrument can perform the earliest detection of brain dysfunction in the elderly. For the past decades, studies have been done for this purpose; however, there has not been any evidence of superiority of a specific screening test. Some argue that, because memory impairment is the earliest clinical feature in AD, performing a more detailed memory examination or incorporating a short-list memory test into a global cognitive assessment may be beneficial for early detection [2]. There are still challenges in performing and interpreting the results of these cognitive assessments that are intended for screening purposes in the elderly, such as the bias possibility due to the influence of level of education on these tests. Adjustments to local normative values are also often warranted [3,4]. Therefore, an ideal screening instrument that is less influenced by age or level of education can be very useful for detection of AD, especially for those living in developing countries.

The aim of this study was to investigate the relationship between the presence of APOE ε4 genotype, a risk factor for AD, and level of education with a verbal memory test (using the Word List Memory Recall Immediate Recall/WLM IR and Saving Score/SS) in the elderly with normal global cognitive assessment (Normal Mini Mental State Examination/MMSE score). The presence of APOE ε4 is linked with an earlier age at onset of AD and therefore was used as an indicator of early possible dementia risk [5].

## 2. Materials and Methods

This was a cross-sectional study performed in subjects ≥60 years old with normal MMSE scores. The MMSE implemented in this study was the Indonesian Version which normative values had been adjusted with age and educational level [3] (See Table S1). Subjects previously participated in preliminary study (baseline cognitive assessment study on quality of life in the elderly living in Kali Anyar, West Jakarta) conducted by the Center of Health Research—Atma Jaya Catholic University of Indonesia between August 2011 and November 2011.

All subjects underwent a standardized structural clinical evaluation including medical history, physical examination and a comprehensive cognitive assessment along with routine blood tests and APOE genetic assessments. APOE assessment was performed using Restriction Fragment Length Polymorphism (RFLP) methods using the Hha I enzyme. The reagent for the extraction was the High Pure Polymerase Chain Reaction (PCR) Template Preparation Kit and the reagent for PCR was Fast Start Taq DNA Polymerase supplied by Roche Applied Biosystem^©^ [6]. Cholesterol level was defined as: High Low Density Level (LDL) ≥130 mg/dL and Low High Density Level (HDL) ≤40 mg/dL [7]. Based on the body mass index (BMI), we defined overweight if BMI >25 kg/m^2^ and normal if BMI <25 kg/m^2^ [8]. There were 105 subjects who met the following inclusion criteria: no previous history of significant head injury/cerebrovascular disease, no consumption of medication that might alter the structure and function of brain, alcoholism, epilepsies, Diabetes Mellitus (history of anti-glycemic agents or Fasting Blood Glucose level ≥126 mg/dL [9]), depression (*Geriatric Depression Scale*/GDS > 4 [10,11]). Each subject was then screened for memory impairment using WLM IR and SS (a neuropsychological test battery included in Consortium to Establish a Registry for Alzheimer Disease/CERAD) [12]. The WLM IR is a free recall memory test reflecting the learning ability for new verbal information; 10 words are presented three times in a different order and the subject is asked to recall as many of the words as possible. The maximum score on each trial is 10 [12]. Delayed recall reflects the ability to recall as many of the 10 words given in the word list memory test after a 5-mins delay. The Saving Score is computed as a percentage, reflecting the relative amount of verbal information retained over the delay interval (delayed recall/WLM IR third trial) × 100% = SS [12]. Memory impairment was defined as WLM IR third trial <8 or SS <80% [12]. This study was approved by the regional ethical committee and written informed consent was obtained from each subject before study onset.

## 3. Results and Discussion

The majority of the subjects were female (68.6%) with a mean age of 65 ± 7.1 years. More than half of the subjects only had formal education <6 years (56.2%). Memory impairment was prominent, comprising more than half of the subjects: WLM IR <8 (81%) and saving score <80% (61%). Overweight was seen in 51.4% of the subjects and the majority had high LDL level (65.7%). Half of the subjects displayed systolic hypertension (43.8%) and diastolic hypertension (41%). APOE ε4 genotype was detected in 24.8% of subjects. There was no significant relationship between age, gender, level of education, hypertension, BMI, LDL and HDL level and memory impairment (*p* > 0.05, see Table 1 and Table 2).

APOE had a significant relationship with WLMIR on bivariate analysis (*p* < 0.05). When the analysis (ANCOVA) was adjusted for age, there was still a significant relationship between APOE and WLMIR (F = 6.17, *p* = 0.015). There was no significant relationship between APOE and SS (Table 2).

This study included elderly subjects who had normal adjusted MMSE scores to age and level of education [3]. Despite these normal MMSE scores, the majority of the subjects (81%) had memory impairment as established from their WLM IR third trial scores. This may imply that WLM IR third trial declines earlier than the MMSE, reflecting MMSE to be a more general examination that represents global cognitive function.

We did not find any significant relationship between level of education with memory impairment (*p* > 0.05). This finding is similar to other previous studies where memory tests were not influenced by level of education, which is unlike the MMSE [3,12]. In a study using CERAD by Indrajaya *et al*. [13] in 192 elderly subjects in Jakarta, they found that MMSE scores were influenced by level of education, however, there was no relationship with word list memory recall, and word list recognition. In another study by Turana *et al.* [3], conducted in 1001 elderly subjects in Jakarta, also showed that the MMSE was very susceptible to level of education. Both studies revealed that WLM IR test was applicable to use in the elderly with low educational backgrounds. This can be considered as an advantage in comparison to MMSE total scores, which are very much influenced by educational level warranting adjustments.

**Table 1:** Memory Impairment (Word List Memory Immediate Recall (WLM IR) <8) with Status of Demographic, Clinical, Laboratory and APOE.

Variable	Memory Impairment	Normal	*p*	Odds Ratio	CI 95%
*Education*					
<6 years	51 (86.4%)	8 (13.6%)	0.17	2.25	0.83–6.08
≥6 years	34 (73.9%)	12 (26.1%)	-	-	-
*Gender*					
Man	30 (90.9%)	3 (9.1%)	0.14	3.09	0.84–11.40
Woman	55 (76.4%)	17 (23.6%)	-	-	-
*Age*					
>65 years old	32 (91.4%)	3 (8.6%)	0.09	3.42	0.93–12.60
≤65 years old	53 (75.7%)	17 (24.3%)	-	-	-
*LDL*					
High	54 (78.3%)	15 (21.7%)	0.48	0.58	0.19–1.75
Normal	31 (86.1%)	5 (13.9%)	-	-	-
*HDL*					
Low	29 (85.3%)	5 (14.7%)	0.60	1.55	0.51–4.70
Normal	56 (78.9%)	15 (21.1%)	-	-	-
*BMI*					
Overweight	45 (83.3%)	9 (16.7%)	0.70	1.38	0.52–3.66
Normal	40 (78.4%)	11 (21.6%)	-	-	-
*Systolic blood pressure*					
Hypertension	38 (82.6%)	8 (17.4%)	0.90	1.21	0.45–3.27
Normal	47 (79.7%)	12 (20.3%)	-	-	-
*Diastolic blood pressure*					
Hypertension	34 (79.1%)	9 (20.9%)	0.88	0.81	0.30–2.17
Normal	51 (82.3%)	11 (17.7%)	-	-	-
*APOE Genotype*					
Positive (ε4 +)	25 (96.2%)	1 (3.8%)	0.02	7.92	1.01–62.38
Negative (ε4 –)	60 (75.9%)	19 (24.1%)	-	-	-
*APOE Genotype*					
APOE +/+	2 (100.0%)	0 (0.0%)	0.07	-	-
APOE −/+	23 (95.8%)	1 (4.2%)	-	-	-
APOE −/−	60 (75.9%)	19 (24.1%)	-	-	-

Chi-Square Test.

In this study, having the ε3 allele was the most frequent, followed by the ε4 allele and ε2 allele. In three separate studies where the subjects were Chinese, Swedish, and Turkish, similar distribution of APOE genotypes with genotype ε3/3 being the most frequent (>50%) were also shown and ε2/2, ε2/4 genotypes were less common (<5%) [14,15,16].

**Table 2:** Memory Impairment (Saving Score <80%) with Status of Demographic, Clinical, Laboratory, and APOE.

Variable	Memory Impairment	Normal	*p*	Odds Ratio	CI 95%
*Education*					
<6 years	37 (62.7%)	22 (37.3%)	0.69	1.18	0.542.61
≥6 years	27 (58.7%)	19 (41.3%)	-	-	-
*Gender*					
Man	20 (60.6%)	13 (39.4%)	1.00	0.98	0.422.23
Woman	44 (61.1%)	28 (38.9%)	-	-	-
*Age*					
>65 years old	23 (65.7%)	12 (34.3%)	0.53	1.36	0.58–3.16
≤65 years old	41 (58.6%)	29 (41.4%)	-	-	-
*LDL*					
High	43 (62.3%)	26 (37.7%)	0.83	1.18	0.522.69
Normal	21 (58.3%)	15 (11.7%)	-	-	-
*HDL*					
Low	19 (55.9%)	15 (44.1%)	0.52	0.73	0.321.68
Normal	45 (63.4%)	26 (36.6%)	-	-	-
*BMI*					
Overweight	33 (61.1%)	21 (38.9%)	1.00	1.01	0.462.22
Normal	31 (60.8%)	20 (39.2%)	-	-	-
*Systolic blood pressure*					
Hypertension	26 (56.5%)	20 (43.5%)	0.43	0.72	0.331.58
Normal	38 (64.4%)	21 (35.6%)	-	-	-
*Diastolic blood pressure*					
Hypertension	23 (53.5%)	20 (46.5%)	0.23	0.59	0.271.31
Normal	41 (66.1%)	21 (33.9%)	-	-	-
*APOE Genotype*					
Positive (ε4 +)	14 (53.8%)	12 (46.2%)	0.48	0.68	0.281.66
Negative (ε4 –)	50 (63.3%)	29 (36.7%)	-	-	-
*APOE Genotype*					
APOE +/+	2 (100%)	0 (0%)	0.26	-	-
APOE −/+	12 (50.0%)	12 (50.0%)	-	-	-
APOE −/−	50 (63.3%)	29 (36.7%)	-	-	-

Chi-square test.

With regards to genetics, our findings indicate a significant relationship between the presence of ε4 and lower WLM IR scores but not with SS scores. This finding is quite interesting and we propose that, because WLMIR assesses learning ability for new verbal information whereas SS reflects delay recall, the presence of ε4 may have an effect on learning ability earlier than delay recall. Nevertheless, further research is required to confirm this finding. There were two subjects who were ε4 allele homozygout and both scored very low WLM IR and SS compared to other subjects. This is supported by Ramakers *et al.* [17], who stated that the APOE ε4 allele was most strongly related to memory learning performance. Although nowadays APOE genotyping has not been routinely performed in the diagnostic work up, many studies have shown that APOE is well-known risk factor for cognitive impairment and an indicator for a more rapid cognitive decline in old age [18]. A study by Fleisher, *et al.* [19] showed that cerebral amyloid deposition, which occurs early in the pathophysiological development of AD, is associated with APOE ε4 carrier status in older healthy control subjects and symptomatic AD patients, and that thus increases with age in older cognitively normal individuals. Amyloid imaging positivity appears to begin near age 56 years in cognitively intact APOE ε4 carriers.

We did not find any relationship between cholesterol levels (LDL and HDL) and memory impairment. Several similar studies investigating the role of plasma lipids or lipid lowering treatments and cognitive function have also reported inconsistent results [20,21,22]. Our study is similar to Gillum *et al.* [23], where they did not find any relationship between HDL level and cognitive function. Yasuno *et al.* [24] also supported a lack of relationship between LDL, triglycerides, and total cholesterol level with cognitive scores. Finally, Elias *et al.* [25] also stated that there was no relationship between cholesterol with amnestic type Mild Cognitive Impairment (aMCI) supporting the view that memory function is not necessarily related to cholesterol levels.

Other risk factors, such as obesity and hypertension, were also not significantly related to cognitive function in our study. Another study revealed a relationship between obesity and cognitive function in male subjects [26,27]. After performing sub-analysis, we did not find any significant relationship between cognitive function and BMI in male subjects. Hypertension remains controversial in its role as a risk factor in a-MCI and dementia. Vascular diseases are known as risk factor for MCI in some studies but some contradicting results also exist [28,29]. Farmer *et al.* [30] demonstrated no relationship between hypertension and cognitive dysfunction. This could support the theory that hypertension is more dominant in non-memory cognitive problems [31].

There are some limitations in our study, such as the cross-sectional design, making it challenging to establish a causal relationship between the variables. Hence, prospective studies should be done in the future for better clinical relevance associations. In this study we found that memory test performance in our low educated subjects was not influenced by level of education. Almost 56% of the subjects only attended formal education for <6 years. This may be different for participants with a larger range in educational levels, including higher level education.

## 4. Conclusions

WLM IR score is lower in elderly people with APOE ε4 despite their normal global cognitive assessment. Screening tests that are less influenced by level of education have an important role in the diagnostic process of cognitive impairment, especially in developing countries, where the majority of elderly people have a low educational background.

## Supplementary Files

Supplementary File 1
