# Supplementary material for: Association between APOE ε4 Genotype and Memory Impairment in Elderly with Normal Global Cognitive Assessment
Source: Diagnostics (Basel). 2015 Dec 15;5(4):615–23. doi: 10.3390/diagnostics5040615 (PMC4728477; doi:10.3390/diagnostics5040615)
Supplement: Supplementary File 1 [file diagnostics-05-00615-s001.pdf]

Supplementary Materials

Table S1. MMSE adjusted with age and educational level (mean and standard deviation).

**Education Level (Years)**	**Age (Years)**			
	**60–65**	**66–70**	**71–75**	**>75**
No Formal Education	17 (4.3)	16 (4.9)	16 (3.5)	14 (4.5)
1–5	23 (4.4)	23 (5.2)	22 (5.1)	21 (4.6)
6–8	26 (2.9)	25 (4.3)	23 (4.4)	23 (4.3)
9–10	27 (2.4)	26 (4.2)	26 (3.4)	24 (6.3)
>12	27 (3.3)	28 (1.7)	27 (2.8)	25 (4.4)

This table is the simplified version of reference number 3; n = 1001 subjects, MMSE = Mini-Mental State Examination.
